# Outlines of the Veins and Lymphatics, with Short Descriptions

**Published:** 1847-11

**Authors:** 


Art. X.—*Outlines of the Veins and Lymphatics, with Short Descriptions. Designed for the Use of Medical Students.* By John Neil, M.D., Demonstrator of Anatomy in the University of Pennsylvania; Lecturer on Anatomy in the Medical Institute of Philadelphia, etc. etc. Philadelphia: E. Barrington and G. D. Haswell. 29 pp. 8vo. 1847.

This well executed little volume belongs to a series, of which we have had occasion to speak more than once in terms of approbation. Concerning the utility of such works there can be no question, and the skill with which the present series has been executed is such as to extort praise from all quarters. Students of medicine will find it a profitable companion.

